# Nanomaterial-Based Dual-Emission Ratiometric Fluorescent Sensors for Biosensing and Cell Imaging

**DOI:** 10.3390/polym13152540

**Published:** 2021-07-31

**Authors:** Yanan Zhang, Dajun Hou, Zelong Wang, Ning Cai, Chaktong Au

**Affiliations:** 1 Hubei Key Laboratory for Processing and Application of Catalytic Materials, College of Chemistry and Chemical Engineering, Huanggang Normal University, Huanggang 438000, China; hdj0068@163.com (D.H.); pctowl@hotmail.com (C.A.); 2 Key Laboratory for Green Chemical Process of Ministry of Education, Hubei Key Laboratory for Novel Reactor and Green Chemistry Technology, School of Chemical Engineering and Pharmacy, Wuhan Institute of Technology, Wuhan 430073, China; wangzelong@hnksac.com

**Keywords:** nanomaterial, ratiometric fluorescent sensor, biosensing, cell imaging

## Abstract

Owing to the unique optophysical properties of nanomaterials and their self-calibration characteristics, nanomaterial-based (e.g., polymer dots (Pdots) quantum dots (QDs), silicon nanorods (SiNRs), and gold nanoparticle (AuNPs), etc.) ratiometric fluorescent sensors play an essential role in numerous biosensing and cell imaging applications. The dual-emission ratiometric fluorescence technique has the function of effective internal referencing, thereby avoiding the influence of various analyte-independent confounding factors. The sensitivity and precision of the detection can therefore be greatly improved. In this review, the recent progress in nanomaterial-based dual-emission ratiometric fluorescent biosensors is systematically summarized. First, we introduce two general design approaches for dual-emission ratiometric fluorescent sensors, involving ratiometric fluorescence with changes of one response signal and two reversible signals. Then, some recent typical examples of nanomaterial-based dual-emission ratiometric fluorescent biosensors are illustrated in detail. Finally, probable challenges and future outlooks for dual-emission ratiometric fluorescent nanosensors for biosensing and cell imaging are rationally discussed.

## 1. Introduction

Fluorescent sensors have garnered increasing attention for biosensing and cell imaging because of their simplicity and high sensitivity. Generally, fluorescent sensors respond to the analyte with enhanced or quenched fluorescence intensity due to the specific sensor–analyte interactions. Comparatively, the “turn-on” switch for a fluorescent sensor has a higher signal-to-noise ratio than a “turn-off” switch [1]. Due to unavoidable interferences from analyte-independent factors, such as environmental fluctuations, instrumental efficiency, local concentration variation of sensors, and the sample matrix, the detection signal arising from the change of intensity at one emission wavelength is usually problematic. To circumvent such a situation, another fluorescence signal is introduced in the design of ratiometric sensors. In this way, these sensors are differentially sensitive to the target for at least two emission wavelengths. The ratios of the emission intensities are then calibrated to indicate the change of target concentration. Similar to other self-calibration analytical methods, ratiometric fluorescence methods can not only improve signal-to-noise ratios but also enable accurate molecular detection and imaging [2,3,4,5,6,7].

A fluorescent sensor usually comprises two components: an analyte recognizer (e.g., DNA, antibody, peptide, or protein) and a signal transducer (e.g., fluorescent nanomaterials and small molecule fluorophores). With the development of nanotechnology, a variety of nanomaterials have been widely used for the fabrication of ratiometric fluorescent biosensors. Compared with fluorescent proteins (FPs) or organic dyes, nanomaterial-based fluorescent sensors possess certain advantages for intracellular applications. The dimensions of these nanomaterials are typically 1–200 nm, favoring entry into live cells by endocytosis, particle bombardment delivery, phagocytosis, pico-injection, or other pathways [8,9,10]. Although FPs are able to report biochemical activities, cell localization, and intracellular small molecule concentration [11,12,13], nanomaterial-based fluorescent biosensors possess an advantage over FP biosensors in that they can probe native cells without gene transfection. Some nanomaterials show outstanding optical properties, and some even exhibit energy/electron-accepting features that can serve as signal-transduction components [14,15,16,17]. With the particular dyes embedded inside a single nanoparticle, the developed nanosensors are expected to have enhanced optical signals in comparison to fluorescent dyes [18]. Additionally, organic dyes are usually encapsulated with nanomaterials to improve their optical stability and biocompatibility [19]. In addition, surface functionalization of the nanomaterials can make them targeted for specific analytes, cells, or even subcellular organelles, thus resulting in effective biosensing and intracellular imaging [20,21,22,23]. Typically, the fabrication of ratiometric fluorescent bionanosensors starts with the preparation of the nanomaterials in one of the following ways: bottom up, top down, or a combination of bottom up and top down that is followed by surface-chemistry modification. The crystal structure, chemical composition, shape, size, and surface properties of the nanomaterial-based ratiometric biosensors play important roles in achieving high performance in fluorescence sensing and cell imaging applications [8,24,25]. 

The sensing mechanisms of the ratiometric fluorescent biosensors for the target analyte are based on strategies including fluorescence resonance energy transfer (FRET), photoinduced electron transfer (PET), inner filter effect (IFE), and intramolecular charge transfer (ICT). Hormozi-Nezhad and coworkers explained the principles of these sensing mechanisms in detail in a recent review [3]. Herein, we focus on the nanomaterial-based ratiometric dual-emission tactics, which can be divided into three categories: (1) nanomaterial-dye(s), (2) hybrid nanomaterials, and (3) single nanomaterials with intrinsic dual emission. Specific examples of these three classes of nanosensor are presented in the subsequent sections.

## 2. Principles for Designing Ratiometric Fluorescent Nanosensors

To achieve ratiometric biosensing or cell imaging, there are two general design approaches for the construction of dual-emission fluorescent nanosensors. One is to design an analyte-sensitive signal and a reference signal insensitive to the analyte. The other is to adopt two analyte-responsive reversible signal changes that allow ratiometric fluorescence.

### 2.1. Ratiometric Fluorescence with One Response Signal

**Figure 1:**
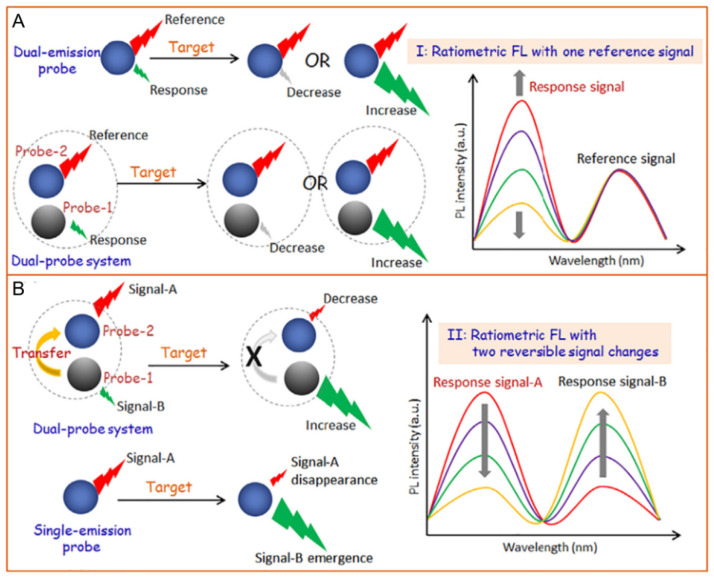
The general categories for fabricating dual-emission ratiometric fluorescent sensors. Ratiometric fluorescence with one reference signal (**A**) or with two reversible signal changes (**B**). Reproduced from [1], with permission from Elsevier, 2019.

As displayed in Figure 1A, the analyte-sensitive signal and the reference signal are from two independent probes. The latter allows the former to be normalized. Basically, a facile way to realize this ratiometric biosensing or cell imaging is through physical mixing of the two probes. However, the requirement for two independent probes can make the methods complicated. For example, uneven distributions of these two probes in cells can result in false imaging results. Single nanoprobes with dual-emission signals show the advantage of eliminating errors related to variations in probe concentration. Preconjugation or preassembly is needed for generating dual-emission signals from a single nanoprobe. This can be achieved via chemical or physical methods. By employing this design strategy, the single nanoprobes become more reliable, further promoting their application in biosensing and cell imaging.

### 2.2. Ratiometric Fluorescence with Two Reversible Signal Changes

Another approach for designing ratiometric dual-emission sensors is illustrated in Figure 1B. Two interrelated analyte-sensitive signals display reversible changes. Generally, one signal can specifically increase in the presence of analytes, while the other decreases. There is an obvious change in the ratio between the two fluorescence signals. A common tactic for this ratiometry is to construct nanoprobes with two signal outputs that can trigger analyte-binding-driven emission phenomena, such as proton transfer, charge transfer, energy transfer, chemical reaction, or physical interactions. The specific binding of probes with analytes can induce the system to produce reversible variations of two signals, thereby allowing for ratiometric fluorescence detection. Various strategies can be applied to induce the signal changes, including antigen–antibody interactions, nucleic acid hybridization, physical absorption/dissociation, and chemical coupling. As an extreme case of this type of ratiometry, stimuli-responsive nanoprobes can be designed to achieve the disappearance of one fluorescence signal, together with the emergence of a new one induced by the analytes. This tactic for ratiometric fluorescence detection is simpler and exhibits a lower background noise, as well as a higher signal-to-background ratio, in comparison to the former. Ratiometric fluorescent sensors with two reversible signal changes can effectively avoid interference from analyte-independent factors and increase their sensitivity. Therefore, they have been widely applied for biosensing and cell imaging during the past decade [26,27,28,29,30,31].

## 3. Nanomaterial-Based Ratiometric Fluorescent Biosensors

Various new nanomaterials have been applied for the construction of ratiometric fluorescent biosensors, such as QDs, carbon dots (CDs), AuNPs, and polymer nanoparticles (PNPs). These nanomaterial-based ratiometric fluorescent biosensors can be used for the precise detection of a variety of targets including pesticides, proteins, nucleic acids, and small molecules. Moreover, they have also been used for direct visualization of the target analyte with the naked eye, as well as in vitro and in vivo imaging [32,33,34,35,36]. The recent developments in the design and application of nanomaterial-based ratiometric biosensors for fluorescence sensing and cell imaging are reviewed in this section. 

### 3.1. QDs-Based Ratiometric Fluorescent Biosensors

**Figure 2:**
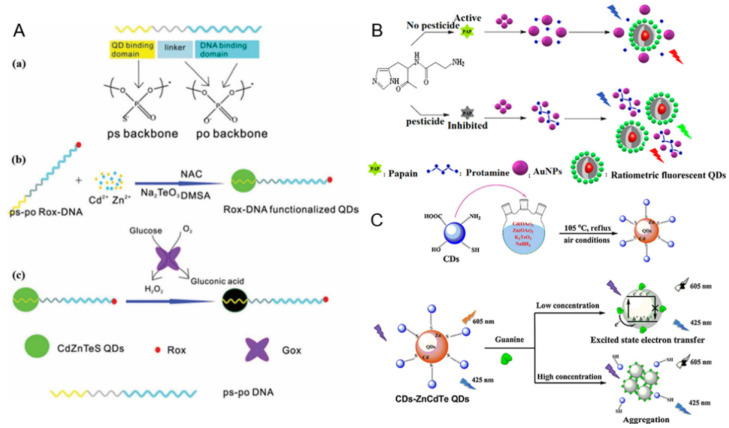
(**A**) Schematic description of (**a**) the structure of ps-po DNA (ps backbone: phosphorothioate linkage, which can bind to QDs due to their high affinity to cadmium; po backbone: phosphate linkage, which is the basic component of DNA.), (**b**) preparation of Rox-DNA functionalized CdZnTeS QDs, and (**c**) H_2_O_2_ detection based on Rox-DNA functionalized CdZnTeS QDs. Reproduced from [42], with permission from the American Chemical Society, 2017. (**B**) Representation of fluorescence detection of CHL through IFE of gold nanoparticles on RF-QDs. Reproduced from [36], with permission from the American Chemical Society, 2020. (**C**) Proposed mechanism of fluorescence sensing guanine by CDs-ZnCdTe QDs. Reproduced from [43], with permission from Elsevier, 2018.

QDs are a class of zero-dimensional nanocrystals with fluorescence properties. The fluorescence properties of QDs stem from their quantum-sized confinement, which occurs when the size of particles is not beyond twice their exciton Bohr radii (about 1–5 nm) [37,38]. However, the quantum confinement is comparatively strong for QDs with sizes smaller than the Bohr radii compared to those with a size larger than the Bohr radii. The first QDs were discovered by Ekimov and Onushchenko in 1981 as copper(I) chloride (CuCl) nanocrystals [39]. Later, there was the more mature development of heavy-metal containing II–VI QDs. These QDs have been widely used in the construction of ratiometric fluorescent biosensors, which is attributed to their excellent properties, such as size-dependent spectra, high quantum yield, broad excitation spectra, narrow emission peaks, and controllable surface functionalization [40,41]. The QD-based ratiometric biosensors are generally classified into three types: (1) dye labeled DNA functionalized QDs sensors, (2) QDs and other nanomaterial hybrid sensors, and (3) intrinsic dual-emitting QDs. He and colleagues reported a facile one-step method to synthesize Rox-DNA functionalized CdZnTeS QDs and used the sensor for ratiometric sensing of glucose in human serum samples [42]. The fluorescence of CdZnTeS QDs can be efficiently quenched by hydrogen peroxide (H_2_O_2_), while the red fluorescence of Rox remains unchanged. As H_2_O_2_ can be generated by the oxidation of glucose through a redox reaction, the ratiometric sensor can be used for sensing of both H_2_O_2_ and glucose (Figure 2A). The established method has achieved a detection limit of 42 nM for glucose and 75 nM for H_2_O_2_. Besides, an obvious color change of the system from green to red can be observed under ultraviolet light irradiation in the presence of blood glucose [42]. Dai and colleagues developed a new biosensor for the sensitive detection of a kind of pesticide named chlorothalonil (CHL), based on the IFE between ratiometric fluorescent QDs (RF-QDs) and AuNPs. The fluorescence of the RF-QDs designed by two different emission CdTe QDs can be quenched by AuNPs. Protamine (PRO) can restore the QD’s fluorescence, due to the interaction between PRO and AuNPs. In the presence of CHL, the hydrolyzation of PRO catalyzed by papain (PAP) can be inhibited effectively, resulting in restored QD fluorescence (Figure 2B). The established sensing platform exhibits a wider linear range (0.34–2320 ng/mL) for CHL than that of single color QDs. Furthermore, the limit of detection for CHL is achieved as low as 0.0017 ng/mL [36]. However, the hybrid nanosensors may encounter the problem of uneven local concentration, which can affect the sensitivity and accuracy. This stimulated the designing of intrinsic dual-emitting nanomaterials as a good alternative. For the first time, Hu and colleagues used fluorescence S-doped CDs as capping ligands to prepare intrinsic dual-emitting ZnCdTe QDs. Compared with the traditional ratiometric sensors prepared by physical methods, the CDs-ZnCdTe QDs were more stable. The proposed sensor could be applied for ratiometric sensing of guanine, as the fluorescence of the QDs can be quenched by the target analyte. The mechanism was probably based on the aggregation of QDs and excited-state electron transfer (Figure 2C) [43]. However, the security risks of Cd-containing QDs have attracted increasing concern. Both high brightness and excellent biocompatibility is required by these kinds of nanomaterial for application in biosensing and cell imaging. Therefore, researchers have turned to exploiting new nanomaterials with low toxicity.

### 3.2. Silicon Nanomaterial-Based Ratiometric Fluorescent Biosensors

Attributed to their unique optical/electronic features, abundant sources, low toxicity, and benign biocompatibility, silicon-based nanomaterials have broad applications in the electronic and biomedicine industries, such as for biosensors, field-effect transistors, drug carriers, and light-emitting diodes [44,45,46]. Among them, fluorescent silicon nanodots (SiNDs) and nanorods have been applied for the design of ratiometric biosensors.

**Figure 3:**
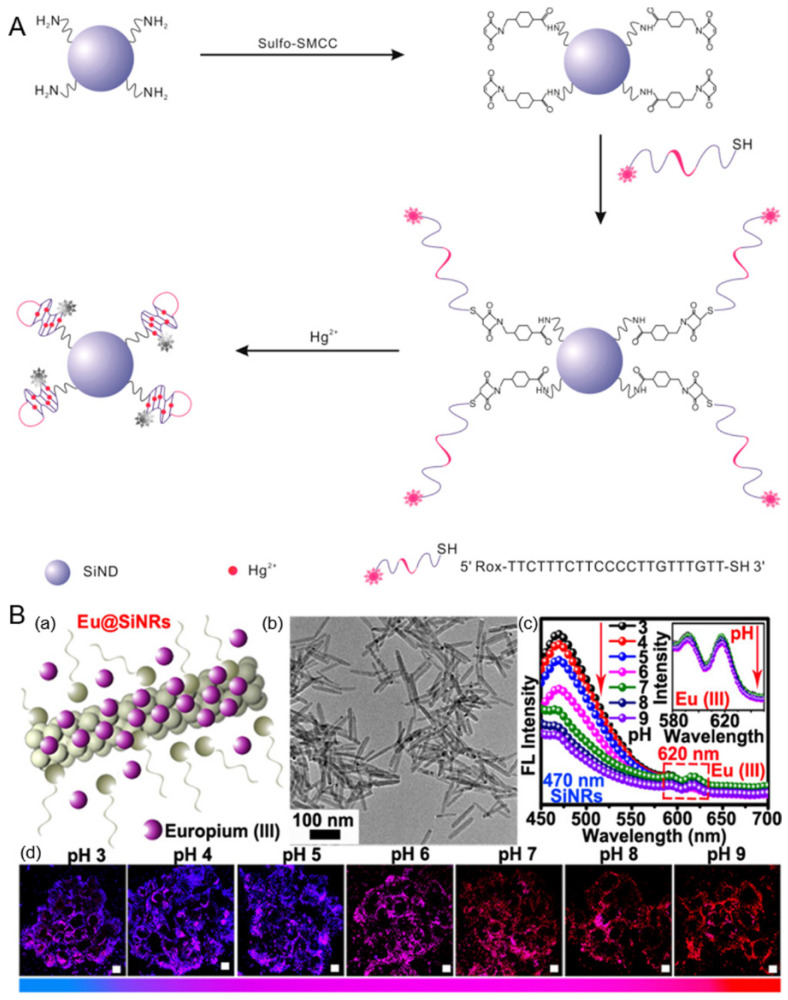
(**A**) Schematic presentation of the preparation of SiND-DNA-Rox and its application for ratiometric detection of Hg^2+^. Reproduced from [23], with permission from the American Chemical Society, 2018. (**B**) (**a**) Schematic of Eu@SiNRs sensor, (**b**) TEM image of Eu@SiNRs, (**c**) fluorescence spectra of Eu@SiNRs in PBS buffers with different pH values under 405 nm excitation, (**d**) a Eu@SiNRs-based pH sensor for ratiometric measurements of cytoplasmic pH in live cells. Reproduced from [56], with permission from the American Chemical Society, 2017.

SiNDs crystals, a class of metal-free QDs, were first reported by Littau et al. in 1992 [47]. Compared with traditional bulk silicon materials, SiNDs exhibit special optical properties due to quantum confining, as well as size and surface effects. At present, the synthesis methods of SiNDs can be divided into two types: bottom-up and top-down. By transmission electron microscopy (TEM) observation, most SiNDs display spherical morphology particles with a diameter of around 2–5 nm [25]. In 2013, by a microwave irradiation “bottom-up” method, Zhong et al. adopted (3-aminopropyl)trimethoxysilane and trisodium citrate as reactants to synthesize highly photostable SiNDs in aqueous solutions [48]. Since then, various strategies have been proposed to prepare water-soluble SiNDs with bioapplications [25]. However, the large-scale preparation and rational functionalization of SiNDs via a simple method for special optical applications remains a challenge. Our group demonstrated that water-soluble SiNDs can be easily prepared at gram scale by the hydrothermal method [20,49]. Then a series of SiNDs-based ratiometric biosensors were constructed for sensing pH, mucin 1, Hg^2+^, DNA, and dipicolinic acid in human serums or the cellular environment [20,21,22,23,50,51]. For example, we developed a ratiometric fluorescent Hg^2+^ sensor by covalent modification of SiND with Hg^2+^-specific 6-carboxyX-rhodamine (Rox)-tagged DNA (Figure 3A). The SiND can quench the red fluorescence of Rox in the presence of the target ions owing to DNA structure change. Meanwhile, the fluorescence of SiND does not alter and serves as the reference signal. This nanosensor was successfully applied for ratiometric imaging of Hg^2+^ in HeLa cells. To date, SiND-associated optical applications have focused on bioimaging [52,53], while relatively little work has touched on their biosensing aspects [54]. The application of SiNDs in biosensing should be further explored. In addition, the integration of biosensing with imaging could enable observations insightful for biochemical analysis [55]. 

He and colleagues prepared europium-doped fluorescent SiNRs (Eu@SiNRs) for ratiometric sensing of intracellular pH for the first time (Figure 3B). Under the excitation of a single wavelength, the fluorescent Eu@SiNRs sensor could simultaneously generate two emission peaks. The fluorescence emission intensity of Eu (III) (620 nm) is invariable with pH changes, while that of SiNR (470 nm) decreases along with the increase of pH, making pH sensing by Eu@SiNRs ratiometrically feasible. The developed sensor has a wide pH response range (pH 3–9) and good biocompatibility, as well as favorable photostability, allowing real-time and long-term measurement of cytoplasmic pH in living HeLa and MCF-7 cells [56]. 

### 3.3. CDs-Based Ratiometric Fluorescent Biosensors

**Figure 4:**
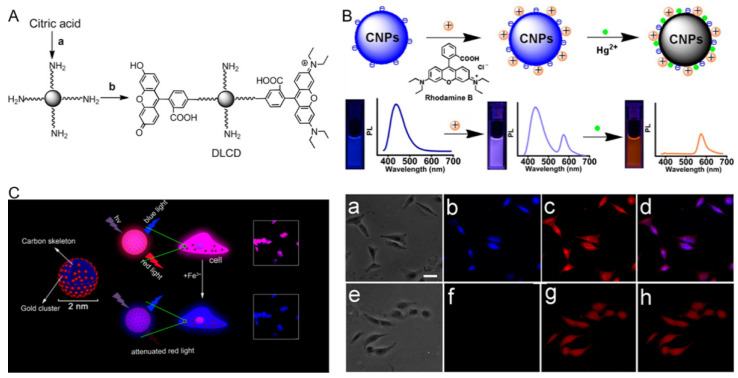
(**A**) Preparation of dual-labeled carbon nanodots (DLCDs). a: TTDDA, 220 °C, 3 h; b: FITC, RBITC, room temperature, overnight. Reproduced from [58], with permission from Wiley, 2012. (**B**) Dual-emission fluorescence sensing of Hg^2+^ based on a CNP–RhB nanohybrid system. Images of A549 cells after being incubated with 200 μL of CNP–RhB nanohybrid solution in the (**a**–**d**) absence and (**e**–**h**) presence of Hg^2+^; (**a**,**e**) bright-field images; (**b**,**f**) blue fluorescence field images; (**c**,**g**) red fluorescence field images; (**d**) the merged images (**b**,**c**); (**h**) the merged images (**f**,**g**). The scale bar is 20 μm. Reproduced from [59], with permission from the American Chemical Society, 2014. (**C**) AuNCs–CD sensor for ratiometric fluorescence imaging of intracellular Fe^3+^. Reproduced from [60], with permission from the American Chemical Society, 2016.

To date, the most widely used carbon nanomaterials are single/multiple walled nanotubes and graphene (oxide), but CDs (e.g., graphene quantum dots and carbon nanodots (CNDs)) have emerged as novel nanomaterials for biosensor construction. CDs display unique properties, such as benign biocompatibility, low cytotoxicity, and high photostability. As an alternative to traditional Cd-containing QDs, CDs with such fascinating features are good candidates for biological applications. Yang and colleagues made a detailed summary of the photoluminescence (PL) mechanism of fluorescent CDs in a review [57]. The CD-based ratiometric fluorescent biosensors can generally be divided into three types: (1) two-dye-linked non-fluorescent CDs, (2) one-dye-modified fluorescent CDs, and (3) CDs and other nanomaterial hybrid sensors. Corresponding typical examples will be introduced later. Shi et al. reported a ratiometric fluorescent pH sensor based on non-fluorescent CNDs for sensing intracellular pH. Owing to their small size (about 5 nm), CNDs can function as a favorable delivery matrix. The pH-insensitive rhotamine B isothiocyanate (RBITC) and pH-sensitive FITC were engrafted onto amino-terminated CNDs via a covalent method (Figure 4A). The intensity of intracellular green fluorescence (FITC) gradually increased with the increase of pH from 6.0 to 8.0, while there was no significant change in the intensity of red fluorescence (RBITC). This ratiometric sensor could be used to monitor the intracellular pH of HeLa cells whose fluctuation is induced by oxidative stress [58]. Nonetheless, nanomaterials are non-fluorescent, and the combination of two fluorescent dyes with CDs is necessary. Thus the synthesis method is usually time-consuming. Lan et al. prepared water-soluble fluorescent carbon nanoparticles (CNPs) having diameters of 15~20 nm and using a microwave-assisted hydrothermal method with trisodium citrate dihydrate and melamine as precursors. The CNPs were combined with rhodamine B (RhB) to form nanohybrid CNP–RhB, which was used for the ratiometric sensing of Hg^2+^ (Figure 4B). Hg^2+^ can completely quench the fluorescence of CNPs through energy or electron transfer process. The fluorescence of RhB was not affected by Hg^2+^ and could be used as a reference signal for internal calibration. The color change from violet to orange can be clearly distinguished with different concentrations of Hg^2+^ by the naked eye under ultraviolet irradiation. The new sensor can effectively identify Hg^2+^ with a detection limit of 42 nM. Additionally, the authors also used fluorescent CNP–RhB for imaging of Hg^2+^ in A549 cells. There was only red fluorescence in the cells, indicating that Hg^2+^ was ingested by the cells and effectively quenched the blue fluorescence of intracellular CNPs [59]. However, RhB is combined with CNPs through electrostatic attraction, which is susceptible to a complicated matrix (e.g., human serum). Zhang et al. constructed a gold nanocluster (AuNC)–CD ratiometric sensor for fluorescence imaging of intracellular Fe^3+^ (Figure 4C). The tiny Au clusters were dispersed in the carbon skeleton by a microwave-assisted method, which integrated the unique properties of CDs and AuNCs. They found that the red fluorescence of AuNCs could be quenched by Fe^3+^, while the fluorescence of the CDs was not altered. The obtained biosensor was successfully applied for dual-color imaging of normal rat osteoblast cells and breast cancer cells, as well as for sensing Fe^3+^ in normal rat osteoblasts cells [60]. Compared with the organic fluorophores, there are no elaborate molecular design and complicated preparation processes for the fabrication of a dual-emission AuNCs–CD nanosensor.

### 3.4. AuNPs-Based Ratiometric Fluorescent Biosensors

**Figure 5:**
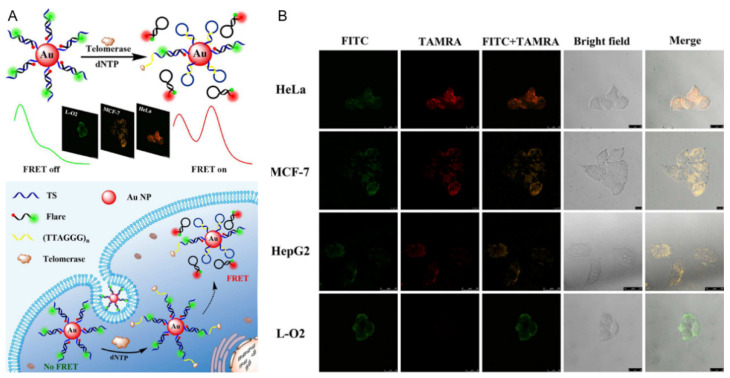
(**A**) Schematic of FRET probe for ratiometric imaging of intracellular telomerase. (**B**) Confocal images of HeLa, MCF-7, HepG2, and L-O2 cells after incubation with 30 μL of probe for 5 h. Reproduced from [67], with permission from the American Chemical Society, 2017.

AuNPs usually exist in the form of colloids in aqueous solutions. Due to their quenching effect and good biocompatibility [17,61,62,63], AuNP-based ratiometric fluorescent sensors have been gradually applied in biochemical analysis in recent years. For example, such sensors can be used to detect telomerase in cells. Telomerase is a complex composed of protein and RNA, which is overexpressed in almost all tumor cells. As a cancer target, telomerase has received considerable attention for the early diagnosis and treatment of cancer [64,65,66]. Xu and colleagues designed a ratiometric FRET nanoflare based on AuNP and the conversion of DNA configuration for the sensing of telomerase in living cells. Telomerase primer sequences (TS) and reporter sequences (Flare) were modified on AuNP to form DNA double strands, in which the 5′ and 3′ ends of Flare were labeled with the fluorescent donor FITC and the receptor carboxylic tetramethylrhodamine (TAMRA), respectively. The detection principle is displayed in Figure 5A. In the absence of telomerase, TS and Flare separated FITC and TAMRA through complementary hybridization, resulting in a low FRET efficiency. Moreover, AuNP can quench the fluorescence of TAMRA. Therefore, only the fluorescence of FITC could be detected. When interacting with telomerase, TS can generate multiple TTAGGG repeats from its 3′ end that are complementary to the corresponding sequence at its 5′ end. Then the Flare is displaced from TS and forms a hairpin structure, narrowing the distance between FITC and TAMRA. The fluorescence intensities of FITC and TAMRA are reduced and enhanced, respectively. Therefore, the fluorescence signal ratio of TAMRA and FITC can be used for quantitative analysis of telomerase. This method was employed to analyze the telomerase in cell lysates, with a detection limit of about 33 HeLa cells. Combined with fluorescence imaging and flow cytometry, the visualization imaging and detection of telomerase activity in different types of cell were realized. As shown in Figure 5B, there is only green fluorescence of FITC in normal liver L-O2 cells, while there is not only green fluorescence of FITC but also red fluorescence of TAMRA in HepG2, MCF-7, and HeLa cancer cells, indicating that this sensor can be used to distinguish cancer cells from normal cells [67]. In addition to quenching the fluorescence of dyes, AuNPs have also been used as a fluorescence quencher for QDs, and the established biosensors could be employed for the quantitative and sensitive detection of pesticides [36,68]. However, there are only a few reports about the construction of AuNP-based ratiometric fluorescent biosensors.

### 3.5. PNPs-Based Ratiometric Fluorescent Biosensors 

**Figure 6:**
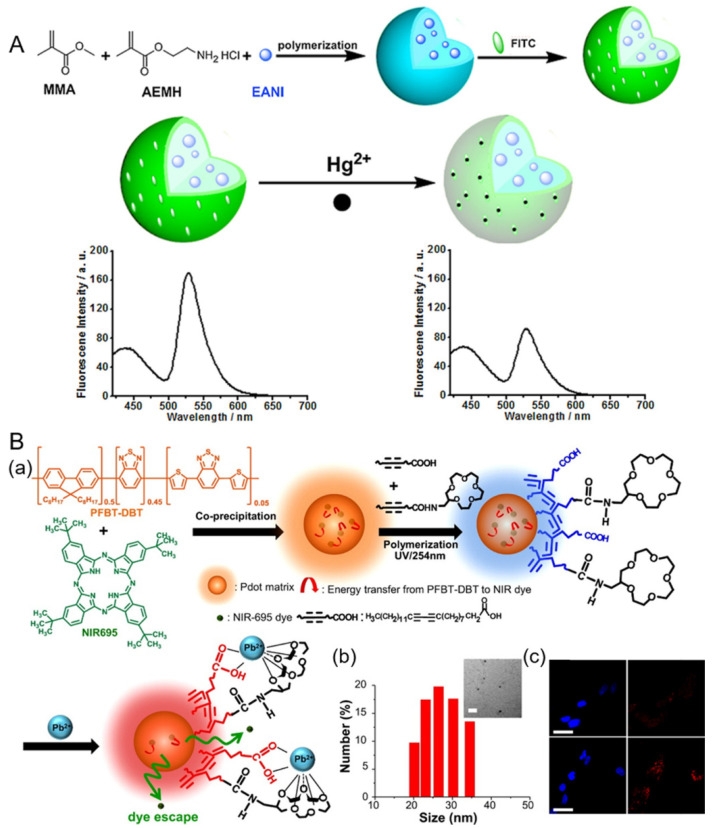
(**A**) Schematic illustration of preparation of a PNP-based ratiometric fluorescent Hg^2+^ sensor. Reproduced from [74], with permission from Elsevier, 2016. (**B**) (**a**) First, semiconducting polymer PFBT-DBT and NIR695 dyes were mixed well in THF and then coprecipitated in water under vigorous sonication to form dye-encapsulated PFBT-DBT Pdots. A mixture of carboxyl- and 15-crown-5-functionalized polydiacetyelenes (PDAs) were then coated onto the surface of the Pdots for subsequent Pb^2+^ sensing. (**b**) The hydrodynamic diameters of PDA-enclosed dye-doped Pdots were measured by DLS. The inset shows their corresponding TEM image. The scale bar represents 100 nm. (**c**) Confocal microscopy imaged HeLa cells labeled by PDA-enclosed NIR695-emdedded Pdots through endocytosis (first row). Images of HeLa cells which were incubated with 20 μM Pb^2+^ for 2 h (second row). Blue fluorescence is from nuclear counterstain Hoechst 34580, and red fluorescence is from Pdots. The scale bars are 30 μm. Reproduced from [78], with permission from the American Chemical Society, 2015.

Fluorescent PNPs possess excellent optical properties, diverse structures, controllable functionalization, and good biocompatibility, and hold great promise for fluorescence detection and bioimaging [69,70]. Wu et al. found that Pdot-bioconjugates are much brighter (~30×) than QD-bioconjugates [71]. Additionally, Pdots also exhibit fast radiative rates and good photostability [72,73]. The PL mechanism of Pdots has been illustrated in the review by Zhu et al. [57]. There are two main types of PNP-based dual-emission ratiometric fluorescent biosensors, which are two-dye-linked non-fluorescent PNPs and one-dye-modified fluorescent PNPs. On the one hand, the fluorescent analyte-sensitive indicators can be covalently bonded to the PNP surface. For example, Wang et al. constructed a PNP-based ratiometric fluorescent Hg^2+^ sensor, using a fluorescein derivative (AEMH-FITC) and 4-ethoxy-9-allyl-1,8-naphthalimide (EANI) as recognition dye and reference fluorescent dye, respectively. The fluorescence of the AEMH-FITC on the surface of PNP can be efficiently quenched by the target Hg^2+^ ions, attributed to PET mechanism, while that of the EANI in the core of PNP remains constant (Figure 6A). The as-prepared sensor shows a quite low detection limit (75 nM) and favorable water dispersibility, and can be used for visualization imaging of Hg^2+^ in living HeLa cells [74]. However, the process for sensor preparation is complicated and time-consuming. On the other hand, the fluorescent analyte-sensitive indicators are either physically trapped or covalently linked within the polymeric nanoparticle matrices [75,76]. There has been ongoing interest in employing polymer or silica nanoparticles as a matrix for the construction of dyes embedded in ratiometric fluorescent biosensors [77,78]. Generally, the leaching of dyes from the matrix is a major concern for many of these sensors [79]. However, this characteristic was utilized by Chan and colleagues to prepare intrinsic fluorescent semiconducting Pdots embedded with near-infrared (NIR) dyes via a facile co-precipitation method for ratiometric detection of Pb^2+^ ions in living HeLa cells (Figure 6B). The high selectivity of Pdots is due to Pb^2+^ chelation on the Pdot surface. After Pb^2+^ chelation, a chromatic change of the complex from blue to red can be observed. Meanwhile, the emission variation of Pdots occurs due to leaching of the encapsulated NIR dyes. The fluorescence intensity of the matrix poly[(9,9-dioctylfluorene)-co-2,1,3-benzothiadiazole-co-4,7-di(thiophen-2-yl)-2,1,3-benzo-thiadiazole] (PFBT-DBT) (650 nm) increased, while the emission intensity of NIR695 (715 nm) gradually decreased with increasing Pb^2+^ concentration. Accordingly, Pb^2+^ ions can be detected by the emission variation and color change of the Pdots [78]. The maximum emission wavelengths of the above two analyte indicators are both not less than 650 nm, which can avoid the influence of cellular autofluorescence to some degree.

From the above statement, we may be curious about why Cd-containing QDs are still used for the construction of biosensors. The great advantage of Cd-containing QDs relies on the property of high quantum yield [80]. However, the heavy metal effects on the environment may hinder their further development. Similarly, although AuNPs can serve as a unique kind of fluorescence quencher, their toxicity is influenced by their surface charge and size [81,82]. New types of environment-friendly nanomaterials, the CDs and SiNDs, have been utilized for sensing extracellular or intracellular molecules in recent years. However, most of them emit short wavelengths, and their quantum yield needs to be improved [25,53,83]. Most PNPs are carbon-based materials that appear to have little pollution [57]. However, the process of preparing them involves organic reagents, which may have an influence on the research staff. Fortunately, nanotechnology and equipment are developing rapidly. We believe that researchers can synthesize CDs and SiNDs with excellent optical properties and prepare PNPs in safe conditions. Herein, it is reasonable to infer that the CDs, SiNDs, and PNPs have the greatest potential for widespread application in biosensing and cell imaging.

## 4. Conclusions

Nanomaterial-based dual-emission fluorescent sensors provide a powerful platform for precise molecular detection and cell imaging, with a high spatiotemporal resolution. As the key components of low-background fluorescence quenchers or fluorescent reporters, nanomaterials play a vital role in ensuring the high sensitivity and imaging contrast of the nanosensors. In addition, the engineerability of nanomaterials through surface modification and structure design enables the intracellular nanosensors to possess good biocompatibility, excellent targeted delivery performance, and superb stability. This review focuses on some of the typical nanomaterials widely used for the fabrication of dual-emission fluorescent biosensors, which may provide guidelines for the development of nanosensors for biosensing and cell imaging applications.

## 5. Challenges and Future Outlooks

Although great progress has been achieved in recent years in the design of nanomaterial-based dual-emission ratiometric fluorescent sensors, there are still several challenges that hinder their development. First, intracellular sensing and imaging applications are impeded by the high autofluorescence background originating from the cells. Most of the currently reported ratiometric fluorescent nanosensors are based on traditional organic dyes, with fluorescence emissions less than 650 nm. To alleviate this situation, NIR dyes are employed for the construction of ratiometric fluorescent nanosensors. However, for many of these ratiometric biosensors, there is usually only one signal output coming from the NIR dye, while the other is still in the short wavelength region (emissions less than 650 nm). Furthermore, the fluorescence of some dyes may be easily affected by the complicated intracellular environment. Therefore, the development of intrinsic dual-emission NIR fluorescent nanomaterials is a good choice to satisfy the requirements of intracellular sensing. Second, ratiometric fluorescent nanosensors for simultaneous analysis of multiple biotargets are attractive. However, there are only a few reports about this kind of nanosensor for the detection of multiple bioanalytes. As such, researchers should devote more efforts to designing ratiometric fluorescent nanosensors for multiplexed biosensing and imaging. Third, simpler design and synthesis schemes should be developed to obtain dual-emission ratiometric bionanosensors with excellent properties, such as high brightness, excellent water solubility, good stability, and low toxicity. Fourth, the application of nanomaterial-based ratiometric fluorescent biosensors for the analysis of targets in single live cells should be explored.

## Data Availability

The data presented in this study are available on request from the corresponding author.

## References

[1] Gui R.J., Jin H., Bu X.N., Fu Y.X., Wang Z.H., Liu Q.Y. (2019). Recent advances in dual-emission ratiometric fluorescence probes for chemo/biosensing and bioimaging of biomarkers. Coordin. Chem. Rev..

[2] Huang X.L., Song J.B., Yung B.C., Huang X.H., Xiong Y.H., Chen X.Y. (2018). Ratiometric optical nanoprobes enable accurate molecular detection and imaging. Chem. Soc. Rev..

[3] Bigdeli A., Ghasemi F., Abbasi-Moayed S., Shahrajabian M., Fahimi-Kashani N., Jafarinejad S., Farahmand Nejad M.A., Hormozi-Nezhad M.R. (2019). Ratiometric fluorescent nanoprobes for visual detection: Design principles and recent advances—A review. Anal. Chim. Acta.

[4] Liu G.X., Ma X.Y., Tang Y.G., Miao P. (2020). Ratiometric fluorescence method for ctDNA analysis based on the construction of a DNA four-way junction. Analyst.

[5] Luo Z.J., Lv T.Y.Z., Zhu K.N., Li Y., Wang L., Gooding J.J., Liu G.Z., Liu B. (2020). Paper-based ratiometric fluorescence analytical devices towards point-of-care testing of human serum albumin. Angew. Chem..

[6] Na M., Zhang S.P., Liu J.J., Ma S.D., Han Y.X., Wang Y., He Y.X., Chen H.L., Chen X.G. (2020). Determination of pathogenic bacteria-Bacillus anthrax spores in environmental samples by ratiometric fluorescence and test paper based on dual-emission fluorescent silicon nanoparticles. J. Hazard. Mater..

[7] Xu Y., Yu H.M., Chudal L., Pandey N.K., Amador E.H., Bui B., Wang L.Y., Ma X.D., Deng S.P., Zhu X.H. (2021). Striking luminescence phenomena of carbon dots and their applications as a double ratiometric fluorescence probes for H_2_S detection. Mater. Today Phys..

[8] Li Q., Liu L., Liu J.W., Jiang J.H., Yu R.Q., Chu X. (2014). Nanomaterial-based fluorescent probes for live-cell imaging. Trac-Trend Anal. Chem..

[9] Li J.J., Cheng F.F., Huang H.P., Li L.L., Zhu J.J. (2015). Nanomaterial-based activatable imaging probes: From design to biological applications. Chem. Soc. Rev..

[10] Maity A.R., Palmal S., Basiruddin S.K., Karan N.S., Sarkar S., Pradhan N., Jana N.R. (2013). Doped semiconductor nanocrystal based fluorescent cellular imaging probes. Nanoscale.

[11] Chu J., Oh Y., Sens A., Ataie N., Dana H., Macklin J.J., Laviv T., Welf E.S., Dean K.M., Zhang F.J. (2016). A bright cyan-excitable orange fluorescent protein facilitates dual-emission microscopy and enhances bioluminescence imaging in vivo. Nat. Biotechnol..

[12] Hanson G.T., McAnaney T.B., Park E.S., Rendell M.E.P., Yarbrough D.K., Chu S.Y., Xi L.X., Boxer S.G., Montrose M.H., Remington S.J. (2002). Green fluorescent protein variants as ratiometric dual emission pH sensors. 1. structural characterization and preliminary application. Biochemistry.

[13] Tang L.T., Zhang S., Zhao Y.F., Rozanov N.D., Zhu L.D., Wu J.H., Campbell R.E., Fang C. (2021). Switching between ultrafast pathways enables a green-red emission ratiometric fluorescent-protein-based Ca^2+^ biosensor. Int. J. Mol. Sci..

[14] Yan F.Y., Zou Y., Wang M., Mu X.L., Yang N., Chen L. (2014). Highly photoluminescent carbon dots-based fluorescent chemosensors for sensitive and selective detection of mercury ions and application of imaging in living cells. Sens. Actuators B.

[15] Ji X.Y., Wang H.Y., Song B., Chu B.B., He Y. (2018). Silicon Nanomaterials for Biosensing and Bioimaging Analysis. Front. Chem..

[16] Singh H., Bamrah A., Bhardwaj S.K., Deep A., Khatri M., Kim K.H., Bhardwaj N. (2021). Nanomaterial-based fluorescent sensors for the detection of lead ions. J. Hazard. Mater..

[17] Li F., Pei H., Wang L.H., Lu J.X., Gao J.M., Jiang B.W., Zhao X.C., Fan C.H. (2013). Nanomaterial-based fluorescent DNA analysis: A comparative study of the quenching effects of graphene oxide, carbon nanotubes, and gold nanoparticles. Adv. Funct. Mater..

[18] Chan Y.H., Wu C., Ye F., Jin Y., Smith P.B., Chiu D.T. (2011). Development of ultrabright semiconducting polymer dots for ratiometric pH sensing. Anal. Chem..

[19] Cai L., Chen Z.Z., Chen M.Y., Tang H.W., Pang D.W. (2013). MUC-1 aptamer-conjugated dye-doped silica nanoparticles for MCF-7 cells detection. Biomaterials.

[20] Zhang Y.N., Hou D.J., Yu X.L. (2020). Facile preparation of FITC-modified silicon nanodots for ratiometric pH sensing and imaging. Spectrochim. Acta Part A.

[21] Zhang Y.N., Guo S., Cheng S.B., Ji X.H., He Z.K. (2017). Label-free silicon nanodots featured ratiometric fluorescent aptasensor for lysosomal imaging and pH measurement. Biosens. Bioelectron..

[22] Zhang Y.N., Guo S., Huang H.Y., Mao G.B., Ji X.H., He Z.K. (2018). Silicon nanodot-based aptasensor for fluorescence turn-on detection of mucin 1 and targeted cancer cell imaging. Anal. Chim. Acta.

[23] Zhang Y.N., Guo S., Jiang Z.R., Mao G.B., Ji X.H., He Z.K. (2018). Rox-DNA functionalized silicon nanodots for ratiometric detection of mercury ions in live cells. Anal. Chem..

[24] Tao X.Q., Peng Y.Y., Liu J.W. (2020). Nanomaterial-based fluorescent biosensors for veterinary drug detection in foods. J. Food Drug Anal..

[25] Song B., He Y. (2019). Fluorescent silicon nanomaterials: From synthesis to functionalization and application. Nano Today.

[26] Liu L., Yang Q.H., Lei J.P., Xu N., Ju H.X. (2014). DNA-regulated silver nanoclusters for label-free ratiometric fluorescence detection of DNA. Chem. Commun..

[27] Wang X.Y., Zhu G.B., Cao W.D., Liu Z.J., Pan C.G., Hu W.J., Zhao W.Y., Sun J.F. (2019). A novel ratiometric fluorescent probe for the detection of uric acid in human blood based on H_2_O_2_-mediated fluorescence quenching of gold/silver nanoclusters. Talanta.

[28] He Y.S., Pan C.G., Cao H.X., Yue M.Z., Wang L., Liang G.X. (2018). Highly sensitive and selective dual-emission ratiometric fluorescence detection of dopamine based on carbon dots-gold nanoclusters hybrid. Sens. Actuators B.

[29] Jin M., Mou Z.L., Zhang R.L., Liang S.S., Zhang Z.Q. (2017). An efficient ratiometric fluorescence sensor based on metal-organic frameworks and quantum dots for highly selective detection of 6-mercaptopurine. Biosens. Bioelectron..

[30] Qi S.J., Liu W.M., Zhang P.P., Wu J.S., Zhang H.Y., Ren H.H., Ge J.C., Wang P.F. (2018). A colorimetric and ratiometric fluorescent probe for highly selective detection of glutathione in the mitochondria of living cells. Sens. Actuators B.

[31] Zhou Z., Wang F.Y., Yang G.C., Lu C.F., Nie J.Q., Chen Z.X., Ren J., Sun Q., Zhao C.C., Zhu W.H. (2017). A ratiometric fluorescent probe for monitoring leucine aminopeptidase in living cells and zebrafish model. Anal. Chem..

[32] Yang Y.L., Mao G.B., Ji X.H., He Z.K. (2020). DNA-templated quantum dots and their applications in biosensors, bioimaging, and therapy. J. Mater. Chem. B.

[33] Guo Z.H., Jiao Y., Du F.F., Gao Y.F., Lu W.J., Shuang S.M., Dong C., Wang Y. (2020). Facile synthesis of ratiometric fluorescent carbon dots for pH visual sensing and cellular imaging. Talanta.

[34] Shi Y.Q., Lin L., Wei Y.Z., Li W.T., Nie P.C., He Y., Feng X.P. (2021). Gold nanoparticles-mediated ratiometric fluorescence aptasensor for ultra-sensitive detection of Abscisic Acid. Biosens. Bioelectron..

[35] Liu C., Lu D.K., You X.R., Shi G.Y., Deng J.J., Zhou T.S. (2020). Carbon dots sensitized lanthanide infinite coordination polymer nanoparticles: Towards ratiometric fluorescent sensing of cerebrospinal Aβ monomer as a biomarker for Alzheimer’s disease. Anal. Chim. Acta.

[36] Sheng E.Z., Lu Y.X., Tan Y.T., Xiao Y., Li Z.X., Dai Z.H. (2020). Ratiometric fluorescent quantum dot-based biosensor for chlorothalonil detection via an inner-filter effect. Anal. Chem..

[37] Chan W.C.W., Nie S.M. (1998). Quantum dot bioconjugates for ultrasensitive nonisotopic detection. Science.

[38] Oliva-Chatelain B.L., Ticich T.M., Barron A.R. (2016). Doping silicon nanocrystals and quantum dots. Nanoscale.

[39] Ekimov A.I., Onushchenko A.A. (1981). Quantum size effect in three-dimensional microscopic semiconductor crystals. Pis’ma Zh. Eksp. Teor. Fiz..

[40] Bruchez M., Moronne M., Gin P., Weiss S., Alivisatos A.P. (1998). Semiconductor nanocrystals as fluorescent biological labels. Science.

[41] Michalet X., Pinaud F.F., Bentolila L.A., Tsay J.M., Doose S., Li J.J., Sundaresan G., Wu A.M., Gambhir S.S., Weiss S. (2005). Quantum dots for live cells, in vivo imaging, and diagnostics. Science.

[42] Mao G.B., Cai Q., Wang F.B., Luo C.L., Ji X.H., He Z.K. (2017). One-step synthesis of Rox-DNA functionalized CdZnTeS quantum dots for the visual detection of hydrogen peroxide and blood glucose. Anal. Chem..

[43] Xu X., He L., Long Y.W., Pan S., Liu H., Yang J.D., Hu X.L. (2019). S-doped carbon dots capped ZnCdTe quantum dots for ratiometric fluorescence sensing of guanine. Sens. Actuators B.

[44] McVey B.F.P., Tilley R.D. (2014). Solution synthesis, optical properties, and bioimaging applications of silicon nanocrystals. Acc. Chem. Res..

[45] Peng F., Su Y.Y., Zhong Y.L., Fan C.H., Lee S.T., He Y. (2014). Silicon nanomaterials platform for bioimaging, biosensing, and cancer therapy. Acc. Chem. Res..

[46] Keshavarz M., Tan B., Venkatakrishnan K. (2018). Multiplex photoluminescent silicon nanoprobe for diagnostic bioimaging and intracellular analysis. Adv. Sci..

[47] Littau K.A., Szajowski P.J., Muller A.J., Kortan A.R., Brus L.E. (1993). A luminescent silicon nanocrystal colloid via a high-temperature aerosol reaction. J. Phys. Chem..

[48] Zhong Y.L., Peng F., Bao F., Wang S.Y., Ji X.Y., Yang L., Su Y.Y., Lee S.T., He Y. (2013). Large-scale aqueous synthesis of fluorescent and biocompatible silicon nanoparticles and their use as highly photostable biological probes. J. Am. Chem. Soc..

[49] Feng Y.L., Liu Y.F., Su C., Ji X.H., He Z.K. (2014). New fluorescent pH sensor based on label-free silicon nanodots. Sens. Actuators B.

[50] Zhang Y.N., Hou D.J., Zhao B.S., Li C.Y., Wang X.Y., Xu L.Y., Long T. (2021). Ratiometric fluorescence detection of DNA based on the inner filter effect of Ru(bpy)_2_(dppx)^2+^ toward silicon nanodots. ACS Omega.

[51] Xu L., Zhang Y.N., Ji X.H., He Z.K. (2019). The ratiometric fluorescent detection of anthrax spore biomarker based on functionalized silicon nanodots. Chem. Pap..

[52] Montalti M., Cantelli A., Battistelli G. (2015). Nanodiamonds and silicon quantum dots: Ultrastable and biocompatible luminescent nanoprobes for long-term bioimaging. Chem. Soc. Rev..

[53] Dasog M., Kehrle J., Rieger B., Veinot J.G.C. (2016). Silicon Nanocrystals and Silicon-Polymer Hybrids: Synthesis, Surface Engineering, and Applications. Angew. Chem. Int. Ed..

[54] Robidillo C.J.T., Wandelt S., Dalangin R., Zhang L.J., Yu H.Y., Meldrum A., Campbell R.E., Veinot J.G.C. (2019). Ratiometric detection of nerve agents by coupling complementary properties of silicon-based quantum dots and green fluorescent protein. ACS Appl. Mater. Inter..

[55] Ru F., Du P.Y., Lu X.Q. (2020). Efficient ratiometric fluorescence probe utilizing silicon particles/gold nanoclusters nanohybrid for “on-off-on” bifunctional detection and cellular imaging of mercury (II) ions and cysteine. Anal. Chim. Acta.

[56] Chu B.B., Song B., Ji X.Y., Su Y.Y., Wang H.Y., He Y. (2017). Fluorescent silicon nanorods-based ratiometric sensors for long-term and real-time measurements of intracellular pH in live cells. Anal. Chem..

[57] Zhu S.J., Song Y.B., Zhao X.H., Shao J.R., Zhang J.H., Yang B. (2015). The photoluminescence mechanism in carbon dots (graphene quantum dots, carbon nanodots, and polymer dots): Current state and future perspective. Nano Res..

[58] Shi W., Li X.H., Ma H.M. (2012). A tunable ratiometric pH sensor based on carbon nanodots for the quantitative measurement of the intracellular pH of whole cells. Angew. Chem. Int. Edit..

[59] Lan M.H., Zhang J.F., Chui Y.S., Wang P.F., Chen X.F., Lee C.S., Kwong H.L., Zhang W.J. (2014). Carbon nanoparticle-based ratiometric fluorescent sensor for detecting mercury ions in aqueous media and living cells. ACS Appl. Mater. Inter..

[60] Zhang L.Y., Wang D.H., Huang H.W., Liu L.F., Zhou Y., Xia X.D., Deng K.Q., Liu X.Y. (2016). Preparation of gold–carbon dots and ratiometric fluorescence cellular imaging. ACS Appl. Mater. Inter..

[61] Lee S., Cha E.J., Park K., Lee S.Y., Hong J.K., Sun I.C., Kim S.Y., Choi K., Kwon I.C., Kim K. (2008). A near-infrared-fluorescence-quenched gold-nanoparticle imaging probe for in vivo drug screening and protease activity determination. Angew. Chem. Int. Ed..

[62] Mayilo S., Kloster M.A., Wunderlich M., Lutich A., Klar T.A., Nichtl A., Kurzinger K., Stefani F.D., Feldmann J. (2009). Long-range fluorescence quenching by gold nanoparticles in a sandwich immunoassay for cardiac troponin T. Nano Lett..

[63] Zhang J., Wang L.H., Zhang H., Boey F., Song S.P., Fan C.H. (2010). Aptamer-based multicolor fluorescent gold nanoprobes for multiplex detection in homogeneous solution. Small.

[64] Hahn W.C., Stewart S.A., Brooks M.W., York S.G., Eaton E., Kurachi A., Beijersbergen R.L., Knoll J.H.M., Meyerson M., Weinberg R.A. (1999). Inhibition of telomerase limits the growth of human cancer cells. Nat. Med..

[65] Harley C.B. (2008). Telomerase and cancer therapeutics. Nat. Rev. Cancer.

[66] Xu Y.C., Goldkorn A. (2016). Telomere and telomerase therapeutics in cancer. Genes.

[67] Yang X.J., Zhang K., Zhang T.T., Xu J.J., Chen H.Y. (2017). Reliable forster resonance energy transfer probe based on structure-switching DNA for ratiometric sensing of telomerase in living cells. Anal. Chem..

[68] Yan X., Li H.X., Han X.S., Su X.G. (2015). A ratiometric fluorescent quantum dots based biosensor for organophosphorus pesticides detection by inner-filter effect. Biosens. Bioelectron..

[69] Chen P.P., Ilyas I., He S., Xing Y.C., Jin Z.G., Huang C.B. (2019). Ratiometric pH sensing and imaging in living cells with dual-emission semiconductor polymer dots. Molecules.

[70] Bao Y.Y., De Keersmaecker H., Corneillie S., Yu F., Mizuno H., Zhang G.F., Hofkens J., Mendrek B., Kowalczuk A., Smet M. (2015). Tunable ratiometric fluorescence sensing of intracellular pH by aggregation-induced emission-active hyperbranched polymer nanoparticles. Chem. Mater..

[71] Wu C.F., Schneider T., Zeigler M., Yu J., Schiro P.G., Burnham D.R., McNeill J.D., Chiu D.T. (2010). Bioconjugation of ultrabright semiconducting polymer dots for specific cellular targeting. J. Am. Chem. Soc..

[72] Verma M., Chan Y.H., Saha S., Liu M.H. (2021). Recent developments in semiconducting polymer dots for analytical detection and NIR-II fluorescence imaging. ACS Appl. Bio Mater..

[73] Men X.J., Wang F., Chen H.B., Liu Y.B., Men X.X., Yuan Y., Zhang Z., Gao D.Y., Wu C.F., Yuan Z. (2020). Ultrasmall semiconducting polymer dots with rapid clearance for second near-infrared photoacoustic imaging and photothermal cancer therapy. Adv. Funct. Mater..

[74] Wang H., Zhang P.S., Chen J., Li Y., Yu M.L., Long Y.F., Yi P.G. (2017). Polymer nanoparticle-based ratiometric fluorescent probe for imaging Hg^2+^ ions in living cells. Sens. Actuators B.

[75] Park E.J., Brasuel M., Behrend C., Philbert M.A., Kopelman R. (2003). Ratiometric optical PEBBLE nanosensors for real-time magnesium ion concentrations inside viable cells. Anal. Chem..

[76] Sun H.H., Scharff-Poulsen A.M., Gu H., Almdal K. (2006). Synthesis and characterization of ratiometric, pH sensing nanoparticles with covalently attached fluorescent dyes. Chem. Mater..

[77] Liu X.J., Zhang N., Bing T., Shangguan D.H. (2014). Carbon dots based dual-emission silica nanoparticles as a ratiometric nanosensor for Cu^2+^. Anal. Chem..

[78] Kuo S.Y., Li H.H., Wu P.J., Chen C.P., Huang Y.C., Chan Y.H. (2015). Dual colorimetric and fluorescent sensor based on semiconducting polymer dots for ratiometric detection of lead ions in living cells. Anal. Chem..

[79] Peng H.S., Stolwijk J.A., Sun L.N., Wegener J., Wolfbeis O.S. (2010). A nanogel for ratiometric fluorescent sensing of intracellular pH values. Angew.Chem. Int. Ed..

[80] Mao G.B., Ma Y.X., Wu G.Q., Du M.Y., Tian S.B., Huang S.Q., Ji X.H., He Z.K. (2021). Novel method of clickable quantum dot construction for bioorthogonal labeling. Anal. Chem..

[81] Xia Q.Y., Li H.X., Xiao K. (2016). Factors affecting the pharmacokinetics, biodistribution and toxicity of gold nanoparticles in drug delivery. Curr. Drug Metab..

[82] Pompa P.P., Vecchio G., Galeone A., Brunetti V., Sabella S., Maiorano G., Falqui A., Bertoni G., Cingolani R. (2011). In Vivo toxicity assessment of gold nanoparticles in Drosophila melanogaster. Nano Res..

[83] Hoan B.T., Tam P.D., Pham V.H. (2019). Green synthesis of highly luminescent carbon quantum dots from lemon juice. J. Nanotech..

